# Associations of different dietary patterns, bone mineral density, and fracture risk among elderly women: the China Osteoporosis Prevalence Study

**DOI:** 10.3389/fendo.2024.1378158

**Published:** 2024-06-12

**Authors:** Nan Zhao, Xiangjun Yin, Lin Chen, Shunyu Tang, Hua Lin, Lu Cui, Xiaolan Jin, Zhongjian Xie, Ning Jiang, Lijia Cui, Wei Yu, Steven R. Cummings, Linhong Wang, Weibo Xia

**Affiliations:** ^1^ Institute of Clinical Medicine, National Infrastructures for Translational Medicine, Peking Union Medical College Hospital, Chinese Academy of Medical Science and Peking Union Medical College, Beijing, China; ^2^ Department of Endocrinology, Key Laboratory of Endocrinology, National Commission of Health, Peking Union Medical College Hospital, Chinese Academy of Medical Sciences and Peking Union Medical College, Beijing, China; ^3^ Division of Elderly Health, National Center for Chronic and Noncommunicable Disease Control and Prevention, Chinese Center for Disease Control and Prevention, Beijing, China; ^4^ Department of Wound Repair and Rehabilitation Medicine, State Key Laboratory of Trauma, Burns and Combined Injury, Daping Hospital, Army Medical University, Chongqing, China; ^5^ Department of Orthopaedics, Nanjing Drum Tower Hospital, The Affiliated Hospital of Nanjing University Medical School, Nanjing, Jiangsu, China; ^6^ Department of Endocrinology, Chengdu Military General Hospital, Chengdu, Sichuan, China; ^7^ Hunan Provincial Key Laboratory of Metabolic Bone Diseases, National Clinical Research Center for Metabolic Diseases, Department of Metabolism and Endocrinology, The Second Xiangya Hospital of Central South University, Changsha, Hunan, China; ^8^ Clinical Cancer Center, National Cancer Center/National Clinical Research Center for Cancer/Cancer Hospital, Chinese Academy of Medical Sciences and Peking Union Medical College, Beijing, China; ^9^ Department of Radiology, Peking Union Medical College Hospital, Chinese Academy of Medical Sciences and Peking Union Medical College, Beijing, China; ^10^ San Francisco Coordinating Center, California Pacific Medical Center Research Institute, San Francisco, CA, United States; ^11^ National Center for Chronic and Non-communicable Disease Control and Prevention, Chinese Center for Disease Control and Prevention, Beijing, China

**Keywords:** dietary pattern, bone mineral density, fracture, Chinese elderly, women

## Abstract

**Objective:**

Despite the fact that China amounts to one-fifth of the world’s population, has a higher proportion of the elderly, and has a higher prevalence of osteoporosis and fracture, limited studies have investigated the association between dietary patterns and bone mineral density (BMD) as well as fracture risk among the elderly Chinese population. We aimed to investigate the association between different dietary patterns and BMD as well as the risk of fractures, and this association may vary between elderly women and men.

**Methods:**

Building upon the China Osteoporosis Prevalence Study, we included 17,489 subjects aged ≥40 years old randomly sampled across 44 counties/districts of 11 provinces or municipalities in China who completed a food frequency questionnaire. BMD was measured by dual x-ray absorptiometry. Vertebral fracture was defined based on lateral spine radiographs using the semi-quantitative technique of Genant.

**Results:**

A diet rich in “carnivorous”, “vegetarian”, “dairy, fruit, and egg” was significantly associated with higher BMD at total hip (TH), femoral neck (FN), and lumbar spine 1–4 (L1–4). Yet, a diet rich in “beverage and fried food” was associated with a lower BMD at the FN and L1–4. High quartiles of the carnivorous diet were associated with 34%–39% reduced risk of clinical fracture in the past 5 years and vertebral fracture. Stronger associations were observed among women. Sensitivity analysis among postmenopausal women presented even stronger positive associations between carnivorous and vegetarian diets and high BMD, as well as between carnivorous diet and reduced risk of fractures.

**Conclusions:**

Our study suggested that a diet rich in meat, vegetables, and dairy, fruit, and eggs might be associated with greater BMD and a lower fracture risk, while beverage and fried foods may be associated with a lower BMD at L1–4, especially among elderly women. These findings are relevant to provide recommendations on dietary nutrition regarding the elderly population at high risk of osteoporosis and fractures, especially postmenopausal women.

## 1 Introduction

Osteoporosis has been considered as an important public health issue due to its increasing prevalence and contribution to morbidity and mortality in the aging population (1–3). Low bone mineral density (BMD), a characteristic feature of osteoporosis, and osteoporosis-related fractures together represent an important public health burden and contribute to socioeconomic disparities (1). Global estimate suggests that the number of hip fractures is expected to increase from 1.26 million in 1990 to 4.5 million by the year 2050 (2, 3). The International Osteoporosis Foundation reports that osteoporosis causes more than 8.9 million fractures annually, with an osteoporosis fracture occurring every 3 s (4) and is estimated to affect 200 million women worldwide (5). In the China Osteoporosis Prevalence Study (COPS), we previously reported that the prevalence of osteoporosis and vertebral fracture in the elderly population (aged 40 years or older) was 20.6% and 9.7% in women and 5.0% and 10.5% in men, respectively (6). As China constitutes one-fifth of the world’s population and a higher proportion of elderly individuals, our previous COPS indicated a higher prevalence of osteoporosis among women and fractures in the elderly in mainland China (6).

Several risk factors have been suggested to be associated with low BMD and risk of fracture, including genetic, endocrine, mechanical, and lifestyle factors such as smoking, alcohol consumption, physical activity, and calcium and vitamin D intake. Early evidence indicates that nutritional factors and dietary patterns play an important role in the development of osteoporosis and the risk of fractures (7–11). In China, few studies have investigated the relationship between dietary factors and the risk of osteoporosis and fractures. Three studies mainly focused on these associations in teenagers or college freshmen aged 11–20 years (12–14). Two studies have investigated dietary patterns associated with BMD (15) and fracture risk (16) among the elderly, but with relatively small sample sizes. However, the results from these studies are inconclusive, and the study participants were randomly selected from one to four cities in mainland China.

In light of the limited research and literature gap regarding the association between dietary patterns and BMD as well as fracture risk among the elderly Chinese population, we analyzed data from the COPS (6), the largest nationwide population-based study conducted in China. The participants enrolled in COPS were randomly sampled from almost all representative regions of mainland China. Building upon the COPS, we aimed to investigate the hypothesis that different dietary patterns are associated with BMD and the risk of fractures and that this association may vary between elderly women and men.

## 2 Materials and methods

### 2.1 Study design and participants

Detailed information regarding the study design was discussed in a previous publication (6). The process of sample size calculation and multistage stratified cluster random sampling across 44 counties/districts of 11 provinces or municipalities in China has been introduced in detail previously. In brief, using a seven-stage stratified cluster random sampling method, the COPS enrolled a representative sample of 20,164 participants aged 20 years or older who had qualified dual-energy x-ray absorptiometry (DXA) measurements from mainland China. After excluding participants without complete dietary information, a total of 17,489 participants aged 40 years or older were included in our study, among whom, 8,423 participants had spine radiographs. All procedures were performed in accordance with protocols approved by the ethical review committee of the Chinese Center for Disease Control and Prevention.

After obtaining written consent from all participants, a standardized, structured questionnaire was used to collect demographic variables, dietary information, and general information such as past medical history, tobacco use, alcohol consumption, occupation and residential histories, and other potential confounders during in-person interviews. Participants were asked about their dietary intake 1 year before the date of disease diagnosis (for cases) or the date of interview (for controls). Alcohol consumption was defined as drinking alcohol at least once a week. Smoking status was defined as those who ever smoked one or more cigarettes per day for at least 1 month. The “Areas” variable was defined as participants’ home addresses being in the southern or northern regions of the Qinling–Huaihe Line (a reference line) in China. Glucocorticoid use (>3 months) was defined as having continuously used glucocorticoid medications for over 3 months.

### 2.2 Measurements of BMD

We transported all participants from the same urban district or rural county to a local hospital with a DXA scanner or to a mobile vehicle with a DXA scanner if one was not available locally. Certified technicians performed DXA on each participant to measure BMD at lumbar spine 1–4 (L1–4), femoral neck (FN), and total hip (TH), using Hologic scanners (Hologic Inc., Walthe, MA, USA) or GE-Lunar scanners (GE Healthcare, Madison, WI, USA). Protocols of cross-calibration among different DXA scanners were introduced in a previous publication (6).

### 2.3 Assessment of fractures

Lateral radiographs of the thoracic and lumbar spine were performed on participants from one randomly selected urban area and one county from each province or municipality. A beam center was set up at T7 for the thoracic spine (covering the level T4 to L1) and L2 for the lumbar spine (covering the level T12 to L5). Two skeletal radiologists assessed vertebral fracture independently based on the semi-quantitative technique of Genant (17), and grade 1 or above fractures were included. The images were reassessed to reach a consensus when disagreements arose (6).

### 2.4 Dietary patterns

Dietary intake information of study participants was assessed using a self-administered, semiquantitative, 17-item food frequency questionnaire (FFQ), which was part of the whole questionnaire/epidemiological survey of the nationwide COPS (6). Each participant recalled the frequency and the usual amount of consumption of each item over the past 12 months. For each food item, five possible frequencies (never, times per day, times per week, times per month, and times per year) and one quantitative (amounts per time: gram for food, milliliter for beverages) response were available. Photographs of food portion sizes were provided to help estimate the amount of food consumption. To avoid unnecessary reduction in statistical power, we assigned a value of 0 servings per year for missing values (*N* = 30), as a missing value for a certain food is likely to indicate 0 consumption (18).

We conducted a principal component analysis of the 17 food items and beverages to identify the study population’s dietary patterns. The principal component analysis was followed by a varimax orthogonal rotation to improve interpretability and minimize correlations between food components. The number of principal components was determined by the eigenvalue-one criterion (also known as the Kaiser criterion, which retains principal components with eigenvalues greater than 1.00), along with the scree test (19). Through these criteria, we were able to retain four principal components, with each component representing a separate, uncorrelated dietary pattern.

The dietary patterns were further ranked by eigenvalue and described as “carnivorous”, “vegetarian”, “dairy, fruit, and egg”, and “beverage and fried food” patterns, through identification of the major foods contributing to the pattern, based on the loading of each food item. Each individual was given a factor score for each dietary pattern. Scores for each dietary pattern were categorized into quartiles, with higher scores representing greater adherence to that dietary pattern (20, 21).

### 2.5 Statistical analysis

COPS described the weighted prevalence of osteoporosis by BMD, the prevalence of vertebral fracture, and a history of clinical fracture in the past 5 years for men and women in five age groups (40–49 years, 50–59 years, 60–69 years, 70–79 years, and ≥80 years) across both urban and rural areas in China. Sample weights were calculated by sampling clusters and post-stratification weights based on the 2010 National Census of China (22), and the final weights were calculated by multiplying the sample weights by the post-stratification weights to represent the general Chinese population.

The mean BMD and its standard deviation (SD) in Chinese men and women, and plotted lumbar spine, FN, and TH by sex were calculated using Svysmooth, a smoothing procedure available in the R survey package 13 (23). The diagnosis of osteoporosis was based on the peak BMD and SD values established for young Chinese men and women aged 20–40 years in the COPS study. According to the WHO diagnostic criteria, *T*-scores = (BMD − Peak BMD of same gender)/(SD of Peak BMD of same gender). Subjects with *T*-scores ≤ −2.5 in any sites (L1–4, FN, or TH) were diagnosed as osteoporosis (24).

Univariate analysis (chi-squared test) was conducted to examine the distributions of selected characteristics for participants. We performed linear regression to investigate the associations between the quartile of a given dietary pattern and BMDs at L1–4, FN, and TH. Unconditional logistic regression models were used to calculate the odds ratios (ORs) and 95% confidence intervals (CIs) for the association between the quartile of a given dietary pattern and risk of overall vertebral fracture, grade 2 or above vertebral fracture, and clinical fracture in the past 5 years (lowest quartile as the reference group). Linear trends were also examined across quartiles in the models. Sex (female and male), age (40–49, 50–59, 60–69, 70–79, and ≥80), BMI (≤18.5, 18.5–23.9, and ≥24.0), education (<college and ≥college), smoking status (never and ever), alcohol consumption (no and yes), residence (rural and urban), area (north and south), family history of fracture (no and yes), serum vitamin D levels (25-hydroxy-vitamin-D), and glucocorticoid use (>3 months) were adjusted for in the multivariable models. We also examined the association between dietary pattern and BMD as well as risk of fracture among men and women and further conducted stratified analyses by sex to investigate whether sex modifies the association between dietary patterns and BMD as well as the risk of fracture. All tests of statistical significance conducted were two-sided and analyses were performed using SAS software (version 9.4; SAS Institute Inc., Cary, North Carolina, USA).

## 3 Results

Of 17,489 participants, there were 7,394 (42.3%) men and 10,095 women with a mean age of 57.5 ± 10.4 and 57.1 ± 9.9 years old, respectively. A total of 725 (4.15%, 725/17,489), 805 (9.56%, 805/8,423), and 331 (3.93%, 331/8,423) participants were diagnosed with clinical fracture in the past 5 years, vertebral fracture, and vertebral fracture grade 2 or above, respectively. The means and SDs of peak BMDs at the TH, FN, and L1–4 are 0.84 ± 0.15 g/cm^2^, 0.74 ± 0.13 g/cm^2^, and 0.92 ± 0.18 g/cm^2^, respectively. **Table 1** shows the distribution of characteristics among men and women who participated in this study. Compared to women, men were more likely to have higher BMD at the TH, FN, and L1–4 (*p* < 0.0001), have a lower prevalence of clinical fracture and vertebral fracture (*p* = 0.0003, 0.002, and 0.21), be older (*p* = 0.003), be a smoker (*p* < 0.0001), be a drinker (*p* < 0.0001), have a higher BMI (*p* = 0.011), have a higher serum vitamin D level (*p* < 0.0001), and reside in a rural area (*p* < 0.0001), and men were less likely to have a higher education level (*p* = 0.0009), glucocorticoid use > 3 months (*p* = 0.036), and calcium supplementation (*p* < 0.0001). Distributions of family history of fracture and residence area in the north or south of China were similar between men and women.

**Table 1:** Distribution of general characteristics of the study subjects by sex.

Characteristics	Men (*N* = 7,394)		Women (*N* = 10,095)		*p* ^1^
Continuous/Categorical	*N*/mean	%/SD	N/mean	%/SD	
BMD (mg/cm^2^) ^2^					
Total hip	0.88	0.14	0.80	0.15	<0.0001
Femoral neck	0.78	0.14	0.71	0.14	<0.0001
L1–L4	0.97	0.17	0.89	0.18	<0.0001
Fractures					
Clinical fracture in the past 5 years	259	3.50	466	4.62	0.0003
Vertebral fracture	421	8.71	384	10.70	0.002
Vertebral fracture ≥ grade 2	130	3.62	201	4.16	0.21
Age (years) ^2^	57.5	10.4	57.1	9.9	0.003
40–49	1,982	26.81	2,668	26.43	<0.0001
50–59	2,110	28.54	3,174	31.44	
60–69	2,335	31.58	3,177	31.47	
70–79	826	11.17	934	9.25	
≥80	141	1.91	142	1.41	
Smoking					
Never	2,856	38.63	9,881	97.90	<0.0001
Ever	4,537	61.37	212	2.10	
Missing	1		2		
Alcohol consumption					
No	5,678	76.80	9,899	98.08	<0.0001
Yes	17,15	23.20	194	1.92	
Missing	1		2		
BMI ^3^					
≤18.5	195	2.64	234	2.32	0.011
18.5–23.9	3,175	42.99	4,551	45.16	
≥24.0	40,15	54.37	52,92	52.52	
Missing	9		18		
Physical activity					
No	5,629	76.1	7,672	76.0	0.84
Yes	1,765	23.9	2,423	24.0	
Family history of fracture					
No	7,085	95.82	9,636	95.45	0.24
Yes	309	4.18	459	4.55	
Education					
< College	7,114	96.21	9,804	97.12	0.0009
≥ College	280	3.79	291	2.88	
Residence					
Urban	3,763	50.89	5,754	57.00	<0.0001
Rural	3,631	49.11	4,341	43.00	
Area					
North	2,729	36.91	3,641	36.07	0.25
South	4,665	63.09	6,554	63.93	
Glucocorticoid use >3 months					
No	7,269	98.32	9,879	97.88	0.036
Yes	124	1.68	214	2.12	
Missing	1		2		
Serum vitamin D (ng/mL) ^2^	29.9	11.0	23.1	8.5	<0.0001
Quartile 1	962	13.08	3,080	30.68	<0.0001
Quartile 2	1,409	19.16	2,872	28.61	
Quartile 3	1,932	26.27	2,477	24.67	
Quartile 4	3,051	41.49	1,610	16.04	
Missing	40		56		
Calcium supplementation					
No	6,451	87.25	7,375	73.06	<0.0001
Yes	943	12.75	2,720	26.94	

^1^Calculated by t-test for continuous variables (BMD, age, and serum vitamin D), Chi-square analysis for other characters.

^2^Mean ± SD.

^3^Weight (kg)/height (m)^2^.

BMD, bone mineral density; BMI, body mass index; SD, standard deviation.

In the principal component analysis (**Supplementary Table 1**), the most prominent dietary pattern (eigenvalue: 2.4, total variance explained: 14.1%) was characterized by the high consumption of carnivorous food. This pattern had the highest positive loadings for poultry, livestock meat, pork, and seafood. The second most prominent pattern represented a high intake of vegetarian food (eigenvalue: 1.4, total variance explained: 8.0%). This pattern had the highest positive loadings for grain, vegetables, bean products, and tubers. The third dietary pattern (eigenvalue: 1.3, total variance explained: 7.7%), which had high levels of dairy, fruit, and egg, featured by milk, yogurt, fruits, and egg. The fourth dietary pattern (eigenvalue: 1.2, total variance explained: 7.2%), which had high intake of beverage and fried food, was featured by fruit juice, soda, fried food, and vegetable juice.

Regression coefficients (β), 95% CIs, *p*-values, and *p* for trends of associations between each dietary pattern and BMD are shown in **Table 2**. Compared to the lowest quartile level, higher quartile levels in “carnivorous”, “vegetarian”, and “dairy, fruit, and egg” patterns were associated with significant increases in BMD at the TH and FN with significant dose–response relationships (*p*_trend_ < 0.0001, 0.0096, and <0.0001 in the above three patterns against with TH; 0.0029, 0.0084, and <0.0001 in the above three patterns against with FN), respectively. Similar positive associations were observed between higher quartile levels in “carnivorous” and “dairy, fruit, and egg” patterns and significant increases in BMD at L1–4 with significant dose–response relationships (*p*_trend_ < 0.0001). However, higher quartile levels in the “beverage and fried food” pattern were associated with significant decreases in BMD at the FN (β = −11.11 and −8.45, *p* = 0.02 and 0.05 for the second and third quartile levels, respectively) and L1–4 (β = −11.52 and −16.80, *p* = 0.03 and 0.002 for the second and third quartile levels, respectively), with no significant dose–response relationship observed.

**Table 2:** Associations between dietary patterns and BMD.

Dietary patterns	Total hip (*N* = 17,405)			Femoral neck (*N* = 17,414)			Lumbar spine 1–4 (*N* = 17,382)		
	β^1^	95% CI	*p*	β^1^	95% CI	*p*	β^1^	95% CI	*p*
Carnivorous diet									
Quartile 1	0.00			0.00			0.00		
Quartile 2	9.72	0.88, 18.55	0.0311	5.96	−2.74, 14.65	0.18	14.35	3.86, 24.85	0.0074
Quartile 3	23.47	14.57, 32.41	<0.0001	19.79	10.89, 28.68	<0.0001	30.95	20.60, 42.40	<0.0001
Quartile 4	17.29	8.12, 26.45	0.0002	10.70	1.98, 19.43	0.0161	31.69	20.79, 42.60	<0.0001
*p_trend_* ^2^	<0.0001			0.0029			<0.0001		
Vegetarian diet									
Quartile 1	0.00			0.00			0.00		
Quartile 2	4.19	−4.68, 13.06	0.35	−1.44	−10.44, 7.57	0.75	3.96	−6.41, 14.34	0.45
Quartile 3	6.10	−1.97, 14.17	0.14	0.89	−7.23, 9.01	0.83	1.17	−9.42, 11.76	0.83
Quartile 4	11.30	2.71, 19.88	0.0098	11.94	3.13, 20.76	0.0079	6.89	−3.80, 17.58	0.21
*p_trend_* ^2^	0.0096			0.0084			0.31		
Dairy, fruit, and egg									
Quartile 1	0.00			0.00			0.00		
Quartile 2	4.77	−3.87, 13.41	0.28	8.31	−0.40, 17.04	0.0615	*5.00*	−*4.85, 14.86*	0.32
Quartile 3	7.13	−1.59, 15.85	0.11	12.70	3.91, 21.50	0.0046	27.30	16.89, 37.71	<0.0001
Quartile 4	19.99	10.79, 29.18	<0.0001	28.30	19.01, 37.59	<0.0001	46.94	35.81, 58.08	<0.0001
*p_trend_* ^2^	<0.0001			<0.0001			<0.0001		
Beverage and fried food									
Quartile 1	0.00			0.00			0.00		
Quartile 2	−7.54	−16.45, 1.37	0.097	−11.11	−20.23, −1.99	0.0169	−11.52	−21.82, −1.23	0.0282
Quartile 3	−3.64	−12.05, 4.75	0.39	−8.45	−16.88, −0.02	0.0494	−16.80	−27.53, −6.07	0.0022
Quartile 4	−0.93	−9.16, 7.30	0.82	−3.14	−11.48, 5.20	0.46	−7.68	−17.87, 2.51	0.1397
*p_trend_* ^2^	0.84			0.76			0.12		

^1^Adjusted for age, sex, BMI, education, smoking, alcohol consumption, residence, area, family history of fracture, serum vitamin D level, and glucocorticoid use >3 months.

^2^Test for trend across quartiles from the linear regression model.

BMD, bone mineral density; BMI, body mass index; CI, confidence interval.

The tests for interaction terms between sex and dietary patterns associated with BMD showed that sex significantly interacts with “carnivorous”, “vegetarian”, and “beverage and fried food” patterns on TH (*p*_interaction_ = <0.0001, 0.02, and 0.02), “carnivorous” and “beverage and fried food” patterns on FN (*p*_interaction_ = <0.0001 and 0.02), and “carnivorous”, “dairy, fruit, and egg”, and “beverage and fried food” patterns on L1–4 (*p*_interaction_ = <0.0001, 0.02, and 0.005). After stratifying by sex, stronger positive associations between “carnivorous”, “vegetarian”, and “dairy, fruit, and egg” patterns and BMD at the TH and FN, as well as stronger inverse associations between “beverage and fried food” pattern and BMD at L1–4 were observed among women (**Table 3**). In contrast, among men, only the highest quartile of “dairy, fruit, and egg” pattern was associated with a significant increase in BMD at the FN and L1–4; the highest quartile of “carnivorous” patterns was associated with a significant increase in BMD at L1–4 (**Supplementary Table 2**). Additionally, those observed significant increases (β) in BMD among men were smaller than those among women and the overall population.

**Table 3:** Associations between dietary patterns and BMD among women.

Dietary patterns	Total hip (*N* = 10,046)			Femoral neck (*N* = 10,055)			Lumbar spine 1–4 (*N* = 10,039)		
	β^1^	95% CI	*p*	β^1^	95% CI	*p*	β^1^	95% CI	*p*
Carnivorous diet									
Quartile 1	0.00			0.00			0.00		
Quartile 2	8.36	−2.88, 19.59	0.14	4.65	−6.63, 15.92	0.42	7.17	−5.69, 20.02	0.27
Quartile 3	23.36	12.85, 33.88	<0.0001	20.23	9.67, 30.79	0.0002	28.70	15.97, 41.44	<0.0001
Quartile 4	25.26	13.59, 36.94	<0.0001	17.74	6.26, 29.21	0.0024	34.46	20.30, 48.63	<0.0001
*p_trend_* ^2^	<0.0001			0.0003			<0.0001		
Vegetarian diet									
Quartile 1	0.00			0.00			0.00		
Quartile 2	−3.04	−14.71, 8.63	0.61	−7.35	−19.13, 4.42	0.22	−6.47	−18.86, 5.91	0.31
Quartile 3	10.12	0.38, 19.88	0.0417	2.21	−7.40, 11.81	0.65	6.30	−6.22, 18.83	0.32
Quartile 4	18.91	8.19, 29.63	0.0005	21.20	10.43, 31.97	0.0001	10.04	−2.50, 22.58	0.12
*p_trend_* ^2^	0.0001			0.0003			0.055		
Dairy, fruit, and egg									
Quartile 1	0.00			0.00			0.00		
Quartile 2	14.44	0.84, 28.04	0.037	17.97	4.01, 31.93	0.0116	15.62	1.87, 29.37	0.026
Quartile 3	9.64	−4.05, 23.32	0.17	16.31	2.17, 30.45	0.0238	34.23	19.58, 48.90	<0.0001
Quartile 4	20.20	6.91, 33.50	0.0029	34.00	20.34, 47.66	<0.0001	47.72	33.55, 61.89	<0.0001
*p_trend_* ^2^	0.0116			<0.0001			<0.0001		
Beverage and fried food									
Quartile 1	0.00			0.00			0.00		
Quartile 2	−5.85	−18.10, 6.41	0.35	−10.67	−23.35, 2.01	0.099	−19.80	−33.37, −6.23	0.0042
Quartile 3	−3.78	−14.11, 6.55	0.47	−8.81	−19.36, 1.75	0.10	−20.81	−34.93, −7.69	0.0017
Quartile 4	7.04	−3.67, 17.76	0.20	6.25	−4.89, 17.39	0.27	−1.12	−14.41, 12.16	0.87
*p_trend_* ^2^	0.19			0.26			0.83		

^1^Adjusted for age, BMI, education, smoking, alcohol consumption, residence, area, family history of fracture, serum vitamin D level, and glucocorticoid use >3 months.

^2^Test for trend across quartiles from linear regression model.

BMD, bone mineral density; BMI, body mass index; CI, confidence interval.

**Table 4** presents the associations between dietary patterns and risk of clinical and vertebral fractures in the overall population. Compared to the lowest quartile of the “carnivorous” pattern, the highest quartile level was associated with a reduced risk of clinical fracture in the past 5 years (OR = 0.63, 95% CI 0.42–0.95, *p*_trend_ = 0.032). A similar protective effect of higher quartile levels of carnivorous pattern on vertebral fracture was observed as well (OR = 0.61, 95% CI 0.43–0.85 for the third quartile; 0.66, 0.45–0.97 for the fourth quartile; *p*_trend_ = 0.017).

**Table 4:** Associations between dietary patterns and risk of fractures.

Dietary patterns	Clinical fracture in the past 5 years (*N* = 17,489, case = 725)		Vertebral fracture (*N* = 8,423, case = 805)		Vertebral fracture grade 2 or above (*N* = 8,423, case = 331)	
	OR^1^	95% CI	OR^1^	95% CI	OR^1^	95% CI
Carnivorous diet						
Quartile 1	1.00		1.00		1.00	
Quartile 2	0.76	0.54, 1.07	0.76	0.55, 1.04	0.69	0.43, 1.11
Quartile 3	0.73	0.51, 1.04	0.61	0.43, 0.85	0.74	0.44, 1.22
Quartile 4	0.63	0.42, 0.95	0.66	0.45, 0.97	0.55	0.26, 1.07
*p_trend_* ^2^	0.032		0.017		0.070	
Vegetarian diet						
Quartile 1	1.00		1.00		1.00	
Quartile 2	1.02	0.75, 1.37	1.00	0.74, 1.36	0.81	0.51, 1.27
Quartile 3	1.22	0.88, 1.65	1.13	0.83, 1.54	0.99	0.60, 1.63
Quartile 4	1.34	0.95, 1.91	1.05	0.73, 1.51	1.13	0.63, 2.05
*p_trend_* ^2^	0.064		0.62		0.62	
Dairy, fruit, and egg						
Quartile 1	1.00		1.00		1.00	
Quartile 2	1.01	0.71, 1.45	1.00	0.73, 1.36	0.76	0.46, 1.24
Quartile 3	1.26	0.91, 1.74	0.84	0.60, 1.17	0.73	0.43, 1.23
Quartile 4	1.21	0.87, 1.69	0.92	0.65, 1.30	0.75	0.43, 1.30
*p_trend_* ^2^	0.12		0.41		0.27	
Beverage and fried food						
Quartile 1	1.00		1.00		1.00	
Quartile 2	1.17	0.81, 1.67	1.04	0.74, 1.45	1.04	0.60, 1.83
Quartile 3	0.97	0.72, 1.32	0.86	0.63, 1.18	0.73	0.44, 1.21
Quartile 4	1.32	0.98, 1.77	1.04	0.77, 1.41	1.30	0.82, 2.07
*p_trend_* ^2^	0.20		0.90		0.52	

^1^Adjusted for age, sex, BMI, education, smoking, alcohol consumption, residence, area, family history of fracture, serum vitamin D level, and, glucocorticoid use >3 months.

^2^Test for trend across quartiles from logistic regression model.

BMI, body mass index; OR, odds ratio; CI, confidence interval.

After the test for interaction terms, the significant interaction was observed only between sex and the “dairy, fruit, and egg” pattern associated with the risk of clinical fracture in the past 5 years (*p*_interaction_ = 0.002), but not vertebral fracture. When conducting stratified analysis by sex, among women (**Table 5**), similar inverse associations were found in higher levels of carnivorous food intake and the risk of clinical and vertebral fractures with a significant dose–response relationship (*p*_trend_ = 0.0083 and 0.013). Moreover, an inverse association was found in the highest level of “carnivorous” and “vegetarian” patterns and the risk of vertebral fracture of grade 2 or above, respectively (OR = 0.45, 95% CI 0.20–0.99, *p*_trend_ = 0.038; 0.48, 0.25–0.93). However, in the “dairy, fruit, and egg” pattern, the third and fourth quartiles increased the risk of clinical fracture (OR = 2.50, 95% CI 1.61–3.88 for the third quartile; 1.99, 1.24–3.17 for the fourth quartile; *p*_trend_ = 0.0002), whereas, among men (**Supplementary Table 3**), only the second quartile of carnivorous food intake was observed to reduce the risk of clinical fracture (OR = 0.48, 95% CI 0.26–0.88), as compared to the first quartile. In addition, the highest quartile of “beverage and fried food” was associated with increased risk of clinical fracture (OR = 1.64, 95% CI 1.06–2.54). No significant association was observed between four dietary patterns and the risk of overall vertebral fracture or vertebral fracture of grade 2 or above in men.

**Table 5:** Associations between dietary patterns and risk of fractures among women.

Dietary patterns	Clinical fracture in the past 5 years (*N* = 10,095, case = 466)		Vertebral fracture (*N* = 4,834, case = 384)		Vertebral fracture grade 2 or above (*N* = 4,834, case = 201)	
	OR^1^	95% CI	OR^1^	95% CI	OR^1^	95% CI
Carnivorous diet						
Quartile 1	1.00		1.00		1.00	
Quartile 2	1.08(0.76, 1.52)		0.78 (0.51, 1.19)		0.66 (0.36, 1.23)	
Quartile 3	0.87 (0.60, 1.27)		0.58 (0.37, 0.90)		0.61 (0.32, 1.16)	
Quartile 4	0.58 (0.39, 0.88)		0.60 (0.37, 0.98)		0.45 (0.20, 0.99)	
*p_trend_* ^2^	0.0083		0.013		0.038	
Vegetarian diet						
Quartile 1	1.00		1.00		1.00	
Quartile 2	1.21 (0.84, 1.72)		0.93 (0.62, 1.40)		0.96 (0.54 1.68)	
Quartile 3	1.11 (0.77, 1.59)		1.27 (0.82, 1.96)		1.35 (0.70, 2.60)	
Quartile 4	1.05 (0.67, 1.60)		0.68 (0.42, 1.09)		0.48 (0.25, 0.93)	
*p_trend_* ^2^	0.82		0.75		0.67	
Dairy, fruit, and egg						
Quartile 1	1.00		1.00		1.00	
Quartile 2	1.41 (0.91, 2.21)		0.93 (0.60, 1.46)		0.75 (0.38, 1.47)	
Quartile 3	2.50 (1.61, 3.88)		0.83 (0.52, 1.32)		0.95 (0.48, 1.84)	
Quartile 4	1.99 (1.24, 3.17)		0.96 (0.59, 1.56)		0.95 (0.45, 2.01)	
*p_trend_* ^2^	0.0002		0.73		0.94	
Beverage and fried food						
Quartile 1	1.00		1.00		1.00	
Quartile 2	0.86 (0.58, 1.29)		1.10 (0.72, 1.68)		1.06 (0.57, 1.99)	
Quartile 3	0.90 (0.62, 1.31)		0.95 (0.60, 1.51)		0.77 (0.38, 1.57)	
Quartile 4	1.06 (0.71, 1.57)		1.03 (0.64, 1.65)		1.28 (0.65, 2.53)	
*p_trend_* ^2^	0.74		0.89		0.71	

^1^Adjusted for age, BMI, education, smoking, alcohol consumption, residence, area, family history of fracture, serum vitamin D level, and, glucocorticoid use >3 months.

^2^Test for trend across quartiles from logistic regression model.

BMI, body mass index; OR, odds ratio; CI, confidence interval.

Finally, we performed a sensitivity analysis among postmenopausal women (**Supplementary Tables 4**, **5**). As compared to the results in women, we observed similar positive associations between “dairy, fruit, and egg” and “beverage and fried food” patterns and BMD at the FN and L1–4, respectively. Particularly, stronger positive associations between carnivorous and vegetarian diets on high BMD at the TH, FN, and L1–4 were found. Additionally, stronger associations between carnivorous diet and reduced risk of clinical fracture, vertebral fracture overall, and vertebral fracture grade 2 or above were also found in postmenopausal women. Similar associations were observed after additional adjustment for physical activity and calcium supplementation (data not shown).

## 4 Discussion

We identified four dietary patterns and observed positive associations between “carnivorous”, “vegetarian”, and “dairy, fruit, and egg” patterns and high BMD at the TH, FN, and L1–4, and inverse associations between the “carnivorous” pattern and risk of both clinical fracture in the past 5 years and vertebral fracture in the overall population. Particularly, stronger associations were seen among women. Additionally, higher intake of beverage and fried food was associated with an increased risk of clinical fracture in men.

To the best of our knowledge, this study is the first to identify dietary patterns within a large, nationwide population-based study in China and to explore these patterns associated with BMD and fracture risk, including differences of these associations between elderly women and men. Prior research in China involving 282 postmenopausal women found that a “cereal grains–fruits” pattern was associated with lower spine and hip BMD, while a “milk–root vegetables” pattern was associated with higher hip BMD (15). Studies in other populations and countries have also observed an association between dietary patterns and BMD, with healthy patterns (prudent/healthy) being associated with higher BMD and unhealthy patterns (Western/unhealthy) being associated with lower BMD. Healthy patterns were characterized by high intakes of fruits, vegetables, low-fat dairy, fish, legumes, high-fiber bread, and poultry, while unhealthy patterns were characterized by red meat, soft drinks, fast food, and sweets (7–10). The current study found similar associations with previous research, with high intakes of vegetarian diets, dairy, fruit, and egg being associated with a higher BMD at the TH, FN, and L1–4. High intakes of beverage and fried food were associated with a lower BMD at the FN and L1–4. Yet, after stratifying by sex, similar or stronger associations were particularly observed in women and postmenopausal women. However, in men, only the highest quartile of dairy, fruit, and egg was positively associated with BMD at the FN and L1–4, suggesting a potential threshold effect or a weaker association in men.

Dairy/milk consumption might have a beneficial effect on bone health for women aged around the menopausal period, which has been demonstrated by some randomized controlled trails (25–28). We enrolled women aged 40 and above, most of whom experienced pre-menopause, menopause, or post-menopause. Early studies indicate that postmenopausal women are at a higher risk of developing osteoporosis and experiencing rapid bone loss due to the decrease in estrogen, which is known to inhibit bone resorption by inducing osteoclast apoptosis (15, 29). The “dairy, fruit, and egg” pattern in the study could be seen as a significant source of calcium, vitamin D, and proteins, all of which are essential for maintaining bone health. Therefore, higher intakes of this pattern might play a significant role in bone health, with gender-specific effects.

Vegetarian diets are rich in various nutrients that are beneficial for bone health, including magnesium, potassium, vitamin C, vitamin K, antioxidant, and anti-inflammatory phytonutrients (30). Evidence suggested that serum magnesium concentration has been shown to be significantly lower in women with osteoporosis than in those with normal BMD (31, 32); potassium intake was associated with higher BMD in men and women (32) and with lower BMD loss in men (33); higher vitamin C intake has been linked to a 44% lower risk of hip fracture (34). Diets rich in phytonutrients may protect bone, but the mechanisms of action have not been conclusively shown (35). However, there was limited evidence suggesting that vegetarians/vegans were more likely to have a lower BMD and a higher risk of developing fractures (36, 37). In the Chinese population. Zeng et al. conducted a matched case–control study suggesting that healthy (fruit and vegetable) or prudent (nuts, mushrooms, algae, and seafood) dietary patterns are associated with a reduced risk of hip fracture in elderly Chinese (16). Similarly, in our COPS, women including those who experienced post-menopause consumed the highest quartile of vegetarian diets and were more likely to have a higher BMD and a lower risk of vertebral fracture. Therefore, this association is still inconclusive. Further research is needed to explore the underlying mechanisms between vegetarian diets and BMD, as well as the risk of fractures.

Our study observed that higher intake of a carnivorous diet was associated with a higher BMD at the TH, FN, and L1–4, as well as a reduced risk of fractures including clinical fracture in the past 5 years and vertebral fracture. When stratified by sex, similar associations were only found in women but not in men. Compared to early studies on the elderly Chinese population, the observations were inconsistent. Zeng et al. have observed that a lower risk of hip fracture was associated with a higher intake of the prudent pattern including seafood, which was similar to us, while a high-fat pattern characterized by red meat, poultry, and animal organ meat was associated with an increased risk. No association was observed between the traditional dietary pattern in China, which includes a high intake of processed meat and fish, and hip fracture risk. Chen et al. found no association between animal protein intake and BMD in postmenopausal Chinese women (15). Unlike early studies, we characterized poultry, livestock meat, pork, and seafood as one dietary pattern—”carnivorous diet”—through principal component analysis. Protein from red meat, seafood, egg, and dairy is an essential source for bone health, contributing to approximately 50% of bone volume and a third of bone mass, and it additionally affects the secretion and action of insulin-like growth factor 1 (IGF-1), which is an orthotropic hormone related to bone formation (9). We also observed a protective effect against fracture risk and lower BMD with a diet rich in vegetarian food among women aged 40 and above, which is seemingly opposite to the carnivorous diet. Recent recommendations suggest a balance between vegetarian and carnivorous diets, emphasizing the importance of proteins, minerals, and vitamins. A chronic vegetarian diet that lacks calcium and certain vitamins might lead to lower BMD (38) and potentially result in osteoporosis and fractures.

Limitation should be considered when interpreting the study results. Firstly, the present study is built upon the COPS; the associations observed may not necessarily indicate causality because of its cross-sectional nature. Yet, the majority of studies developed to elucidate the association between BMD or fracture risk and dietary patterns had a cross-sectional design (9). COPS is the largest nationwide, population-based study of BMD, the prevalence of osteoporosis, and the prevalence of fracture in the Chinese mainland population, considered as a good representative of the general Chinese population. With a nearly 100% participation rate, the difference among regions and other possible confounding factors were likely minimized. Additionally, we collected detailed information on various potential confounding factors and controlled them in the models. Despite these efforts, potential residual confounding from unknown or uncollected variables cannot be completely ruled out. Compared with previous studies investigating the dietary pattern and BMD or fractures in China (15, 16), we included a large sample size (*N* = 17,489) of elderly Chinese population, which allowed exploration of the differences in associations between dietary patterns and BMD status at different locations and risk of different fracture types, as well as stratified associations by sex and postmenopausal status. Secondly, the dietary information used in the study was collected through a self-administered, semiquantitative FFQ during the last 12 months, and thus susceptible to recall bias; most of previous studies that investigated this association, however, utilized FFQ to obtain dietary information (7, 8). We also adjusted factors that were susceptible to confound the results due to dietary recall, such as alcohol consumption, education, BMI, residence (urban and rural), area (north and south), serum vitamin D level, calcium supplementation, and glucocorticoid use. Thirdly, the study identified four dietary patterns based on 17 items, which might not fully represent all food categories. However, the four dietary patterns we selected accounted for 37% of the total variability in the original food variables. Further prospective longitudinal studies are warranted to confirm and examine the association with comprehensive dietary information in the Chinese population.

**Figure 1:**
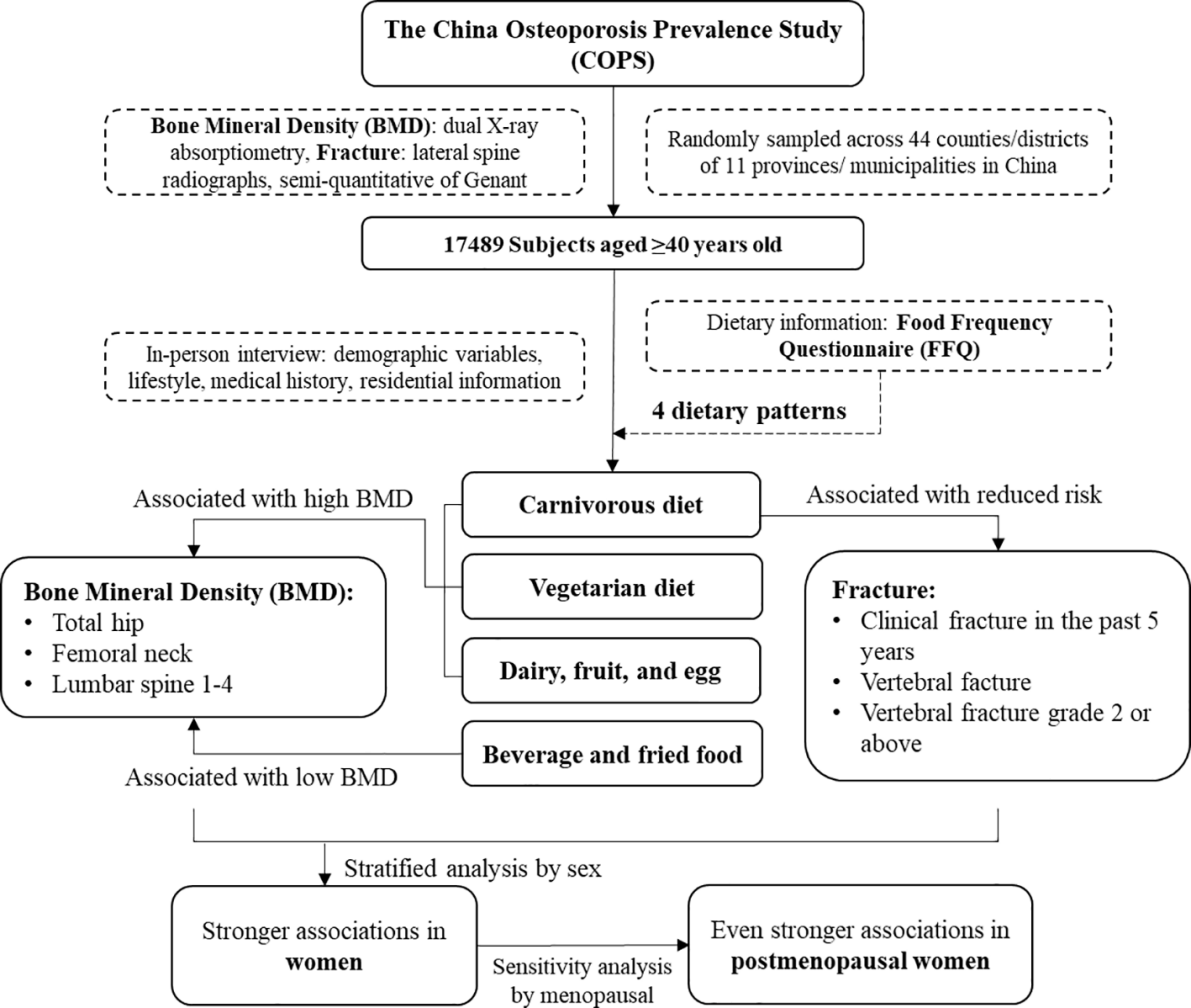
Flowchart summarizing the main findings.

In conclusion, our study suggested that diet patterns rich in meat, vegetables, and dairy, fruit, and eggs might be associated with higher BMD and lower fracture risk, especially among women aged 40 years or older, while a dietary pattern high in beverage and fried foods may be associated with lower BMD and an increased risk of fractures (**Figure 1**). These findings are relevant to provide recommendations on dietary nutrition regarding the elderly population, especially women around the menopausal period, who are at a higher risk of osteoporosis and fractures due to factors such as estrogen decline and age-related bone loss, underscoring the need for gender-specific dietary advice to support optimal bone health (39). Future large studies with sufficient dietary information are necessary to replicate and confirm the observed associations.

## Data availability statement

The raw data supporting the conclusions of this article will be available from the corresponding author upon reasonable request.

## Ethics statement

The studies involving humans were approved by the ethical review committee of Chinese Center for Disease Control and Prevention. The studies were conducted in accordance with the local legislation and institutional requirements. The participants provided their written informed consent to participate in this study.

## Author contributions

NZ: Data curation, Formal analysis, Investigation, Writing – original draft, Writing – review & editing. XY: Data curation, Investigation, Methodology, Supervision, Writing – review & editing. LinC: Investigation, Writing – review & editing. ST: Investigation, Writing – review & editing. HL: Investigation, Writing – review & editing. LuC: Investigation, Writing – review & editing. XJ: Investigation, Writing – review & editing. ZX: Investigation, Writing – review & editing. NJ: Data curation, Investigation, Writing – review & editing. LijC: Data curation, Investigation, Writing – review & editing. WY: Investigation, Writing – review & editing. SC: Writing – review & editing. LW: Funding acquisition, Investigation, Project administration, Supervision, Writing – review & editing. WX: Funding acquisition, Investigation, Project administration, Supervision, Writing – review & editing.
