# Supplementary material for: Associations of different dietary patterns, bone mineral density, and fracture risk among elderly women: the China Osteoporosis Prevalence Study
Source: Front Endocrinol (Lausanne). 2024 Jun 12;15:1378158. doi: 10.3389/fendo.2024.1378158 (PMC11199729; doi:10.3389/fendo.2024.1378158)
Supplement: Supplementary file 1 [file Table_1.docx]

| Supplemental Table 1. Factor loadings of the four major dietary patterns identified by principal component analysis. | | | | | | | | | | | |
| --- | --- | --- | --- | --- | --- | --- | --- | --- | --- | --- | --- |
| Carnivorous diet | |  | Vegetarian diet | |  | Dairy, fruit, and egg | |  | Beverage and fried food | |  |
| Food items | Factor loading |  | Food items | Factor loading |  | Food items | Factor loading |  | Food items | Factor loading |  |
| Poultry | 0.48 |  | Grain | 0.46 |  | Milk | 0.46 |  | Fruit juice | 0.50 |  |
| Livestock meat | 0.38 |  | Vegetables | 0.33 |  | Yogurt | 0.45 |  | Soda | 0.49 |  |
| Pork | 0.36 |  | Bean products | 0.33 |  | Fruits | 0.32 |  | Fried food | 0.29 |  |
| seafood | 0.35 |  | Tubers | 0.31 |  | Egg | 0.19 |  | Vegetable juice | 0.26 |  |

| Supplemental Table 2. Associations Between Dietary Patterns and BMD Among Men | | | | | | | | | | | | |
| --- | --- | --- | --- | --- | --- | --- | --- | --- | --- | --- | --- | --- |
| Dietary patterns | | Total hip (N=7359) | | |  | Femoral neck (N=7359) | | |  | Lumbar spine 1-4 (N=7343) | | |
|  |  | β^1^ | 95%CI | *P* |  | β^1^ | 95%CI | *P* |  | β^1^ | 95%CI | *P* |
| *Carnivorous diet* | |  |  |  |  | | |  |  |  |  |  |
|  | Quartile1 | 0.00 |  |  |  | 0.00 |  |  |  | 0.00 |  |  |
|  | Quartile2 | 1.22 | -12.14, 14.57 | *0.86* |  | 0.21 | -12.63, 13.05 | 0.97 |  | 7.08 | -9.19, 23.36 | *0.39* |
|  | Quartile3 | 12.16 | -2.29, 26.62 | *0.099* |  | 10.87 | -3.49, 25.23 | 0.14 |  | 14.95 | -3.07, 32.98 | *0.10* |
|  | Quartile4 | 6.21 | -7.73, 20.16 | *0.38* |  | 2.08 | -10.91, 15.07 | 0.75 |  | 20.35 | 4.33, 36.37 | *0.013* |
|  | *P_trend_* ^2^ | *0.24* | | |  | *0.56* | | |  | *0.0072* | | |
| *Vegetarian diet* | |  |  |  |  |  | |  |  |  |  |  |
|  | Quartile1 | 0.00 |  |  |  | 0.00 |  |  |  | 0.00 |  |  |
|  | Quartile2 | 4.96 | -8.45, 18.36 | *0.47* |  | -0.18 | -14.12, 13.75 | 0.98 |  | 4.33 | -12.03, 20.69 | *0.60* |
|  | Quartile3 | -2.23 | -14.70, 10.25 | *0.73* |  | -3.64 | -16.92, 9.64 | 0.59 |  | -10.05 | -26.03, 5.93 | *0.22* |
|  | Quartile4 | 0.16 | -12.54, 12.85 | *0.98* |  | 1.67 | -12.06, 15.40 | 0.81 |  | -3.47 | -19.44, 12.51 | *0.67* |
|  | *P_trend_* ^2^ | *0.71* | | |  | *0.90* | | |  | *0.33* | | |
| *Dairy, fruit, and egg* | | |  |  |  | | |  |  |  |  |  |
|  | Quartile1 | 0.00 |  |  |  | 0.00 |  |  |  | 0.00 |  |  |
|  | Quartile2 | -3.92 | -14.96, 7.12 | *0.49* |  | -0.17 | -11.38, 11.04 | 0.98 |  | -3.47 | -16.64, 9.70 | *0.61* |
|  | Quartile3 | -1.05 | -12.49, 10.39 | *0.86* |  | 5.55 | -5.90, 17.00 | 0.34 |  | 10.47 | -3.31, 24.25 | *0.14* |
|  | Quartile4 | 8.99 | -4.65, 22.62 | *0.20* |  | 14.64 | 0.82, 28.46 | *0.038* |  | 29.87 | 13.36, 46.38 | 0.0004 |
|  | *P_trend_* ^2^ | *0.37* | | |  | *0.050* | | |  | *0.001* | | |
| *Beverage and fried food* | | |  |  |  | | |  |  |  |  |  |
|  | Quartile1 | 0.00 |  |  |  | 0.00 |  |  |  | 0.00 |  |  |
|  | Quartile2 | -8.04 | -20.61, 4.53 | *0.21* |  | -10.68 | -23.53, 2.16 | *0.10* |  | 0.32 | -14.27, -14.92 | *0.97* |
|  | Quartile3 | -1.37 | -14.22, 11.47 | *0.83* |  | -6.85 | -19.76, 6.06 | *0.30* |  | -10.01 | -25.81, -5.78 | *0.21* |
|  | Quartile4 | -3.90 | -15.70, 7.91 | *0.52* |  | -7.89 | -19.90, 4.12 | *0.20* |  | -5.21 | -19.33, -8.90 | *0.47* |
|  | *P_trend_* ^2^ | *0.82* | | |  | *0.36* | | |  | *0.29* | | |
| ^1^ Adjusted for age, BMI, education, smoking, alcohol consumption, residence, area, family history of fracture, serum vitamin D level, and glucocorticoid use >3 months | | | | | | | | | | | | |
| ^2^ Test for trend across quartiles from linear regression model  Abbreviations: BMD, bone mineral density; BMI, body mass index; CI, confidence intervals | | | | | | | | | | | | |

| Supplemental Table 3. Associations Between Dietary Patterns and Risk of Fractures Among Men | | | | | | | | | | | | | | | | | | |  |  |  |  |
| --- | --- | --- | --- | --- | --- | --- | --- | --- | --- | --- | --- | --- | --- | --- | --- | --- | --- | --- | --- | --- | --- | --- |
| Dietary patterns | | |  | Clinical fracture in the past 5 years (N=7394, case=259) | | |  | | Vertebral fracture (N=3589, case=421) | | | | |  | Vertebral fracture grade 2 or above (N=3589, case=130) | | | |  |  |  |  |
|  |  |  |  | OR^1^ | | 95%CI |  | | OR^1^ | | 95%CI | | |  | OR^1^ | | 95%CI | |  |  |  |  |
| *Carnivorous diet* | | |  | | | |  | | | | | | |  |  | |  | |  |  |  |  |
|  | | Quartile1 |  | 1.00 | |  |  | | 1.00 | |  | | |  | 1.00 | |  | |  |  |  |  |
|  | | Quartile2 |  | 0.48 (0.26, 0.88) | | |  | | 0.78 (0.50, 1.23) | | | | |  | 0.82 (0.42, 1.60) | | | |  |  |  |  |
|  | | Quartile3 |  | 0.61 (0.34, 1.09) | | |  | | 0.71 (0.44, 1.15) | | | | |  | 1.09 (0.55, 2.17) | | | |  |  |  |  |
|  | | Quartile4 |  | 0.57 (0.31, 1.06) | | |  | | 0.75 (0.44, 1.29) | | | | |  | 0.67 (0.25, 1.89) | | | |  |  |  |  |
|  | | *P_trend_* ^2^ |  | *0.25* | | |  | | *0.33* | | | | |  | *0.58* | | | |  |  |  |  |
| *Vegetarian diet* | | |  | | | |  | | | | | | |  |  | |  | |  |  |  |  |
|  | | Quartile1 |  | 1.00 | |  |  | | 1.00 | |  | | |  | 1.00 | |  | |  |  |  |  |
|  | | Quartile2 |  | 0.83 (0.47, 1.45) | | |  | | 1.11 (0.70, 1.74) | | | | |  | 0.64 (0.30 1.34) | | | |  |  |  |  |
|  | | Quartile3 |  | 1.28 (0.76, 2.15) | | |  | | 1.09 (0.70, 1.69) | | | | |  | 0.64 (0.32, 1.31) | | | |  |  |  |  |
|  | | Quartile4 |  | 1.57 (0.92, 2.67) | | |  | | 1.32 (0.79, 2.21) | | | | |  | 1.41 (0.62, 3.24) | | | |  |  |  |  |
|  | | *P_trend_* ^2^ |  | *0.037* | | |  | | *0.32* | | | | |  | *0.35* | | | |  |  |  |  |
| *Dairy, fruit, and egg* | | | | | | |  | | | | | | |  |  | |  | |  |  |  |  |
|  | | Quartile1 |  | 1.00 | |  |  | | 1.00 | |  | | |  | 1.00 | |  | |  |  |  |  |
|  | | Quartile2 |  | 0.94 (0.58, 1.53) | | |  | | 1.07 (0.71, 1.62) | | | | |  | 0.83 (0.43, 1.62) | | | |  |  |  |  |
|  | | Quartile3 |  | 0.76 (0.47, 1.22) | | |  | | 0.90 (0.57, 1.41) | | | | |  | 0.60 (0.28, 1.30) | | | |  |  |  |  |
|  | | Quartile4 |  | 0.93 (0.55, 1.58) | | |  | | 0.95 (0.59, 1.52) | | | | |  | 0.60 (0.29, 1.22) | | | |  |  |  |  |
|  | | *P_trend_* ^2^ |  | *0.46* | | |  | | *0.69* | | | | |  | *0.13* | | | |  |  |  |  |
| *Beverage and fried food* | | | | | | |  | | | | | | |  |  | |  | |  |  |  |  |
|  | | Quartile1 |  | 1.00 | |  |  | | 1.00 | |  | | |  | 1.00 | |  | |  |  |  |  |
|  | | Quartile2 |  | 1.62 (0.93, 2.74) | | |  | | 0.98 (0.59, 1.62) | | | | |  | 1.02 (0.38, 2.79) | | | |  |  |  |  |
|  | | Quartile3 |  | 1.09 (0.66, 1.82) | | |  | | 0.75 (0.49, 1.15) | | | | |  | 0.68 (0.34, 1.35) | | | |  |  |  |  |
|  | | Quartile4 |  | 1.64 (1.06, 2.54) | | |  | | 0.99 (0.67, 1.46) | | | | |  | 1.24 (0.68, 2.27) | | | |  |  |  |  |
|  | | *P_trend_* ^2^ |  | *0.18* | | |  | | *0.74* | | | | |  | *0.68* | | | |  |  |  |  |
| ^1^ Adjusted for age, BMI, education, smoking, alcohol consumption, residence, area, family history of fracture, serum vitamin D level, and glucocorticoid use >3 months | | | | | | | | | | | | | | | | | | |  |  |  |  |
| ^2^ Test for trend across quartiles from logistic regression model  Abbreviations: BMI, body mass index; OR, odds ratios; CI, confidence intervals | | | | | | | | | | | | | | | |  | | |  |  |  |  |
| Supplemental Table 4. Associations Between Dietary Patterns and BMD Among Postmenopausal Women | | | | | | | | | | | | | | | | | | | | | | |
| Dietary patterns | | | | Total hip (N=6649) | | | | | |  | | Femoral neck (N=6654) | | | | | |  | | L1-L4 (N=6639) | | |
|  |  |  |  | β^1^ | 95%CI | | | *P* | |  | | β^1^ | 95%CI | | | | *P* |  | | β^1^ | 95%CI | *P* |
| *Carnivorous diet* | | | |  |  | | |  | |  | | | | | | |  |  | |  |  |  |
|  | Quartile1 | | | 0.00 |  | | |  | |  | | 0.00 |  | | | |  |  | | 0.00 |  |  |
|  | Quartile2 | | | 26.48 | 11.66, 41.31 | | | *0.0005* | |  | | 20.36 | 5.35, 35.37 | | | | *0.0079* |  | | 10.23 | -6.17, 26.63 | *0.22* |
|  | Quartile3 | | | 62.36 | 47.74, 76.99 | | | *<.0001* | |  | | 51.05 | 36.30, 65.80 | | | | *<.0001* |  | | 47.90 | 30.44, 65.36 | *<.0001* |
|  | Quartile4 | | | 69.98 | 53.86, 86.09 | | | *<.0001* | |  | | 51.58 | 36.07, 67.08 | | | | *<.0001* |  | | 58.33 | 39.02, 77.64 | *<.0001* |
|  | *P_trend_* ^2^ | | | *<.0001* | | | | | |  | | *<.0001* | | | | | |  | | *<.0001* | | |
| *Vegetarian diet* | | | |  |  | | |  | |  | |  | | | | |  |  | |  |  |  |
|  | Quartile1 | | | 0.00 |  | | |  | |  | | 0.00 |  | | | |  |  | | 0.00 |  |  |
|  | Quartile2 | | | 5.68 | -9.78, 21.14 | | | *0.47* | |  | | -2.38 | -17.64, 12.87 | | | | *0.76* |  | | 1.37 | -14.60, 17.35 | *0.87* |
|  | Quartile3 | | | 24.99 | 10.57, 39.42 | | | *0.0007* | |  | | 16.51 | 2.77, 30.25 | | | | *0.02* |  | | 13.04 | -4.29, 30.37 | *0.14* |
|  | Quartile4 | | | 28.41 | 12.87, 43.95 | | | *0.0003* | |  | | 31.13 | 15.50, 46.76 | | | | *<.0001* |  | | 23.51 | 5.45, 41.57 | *0.011* |
|  | *P_trend_* ^2^ | | | *0.0001* | | | | | |  | | *<.0001* | | | | | |  | | *0.0075* | | |
| *Dairy, fruit, and egg* | | | | |  | | |  | |  | | | | | | |  |  | |  |  |  |
|  | Quartile1 | | | 0.00 |  | | |  | |  | | 0.00 |  | | | |  |  | | 0.00 |  |  |
|  | Quartile2 | | | 5.57 | -10.52, 21.66 | | | *0.50* | |  | | 13.13 | -2.85, 29.10 | | | | *0.11* |  | | 10.65 | -6.20, 27.51 | *0.22* |
|  | Quartile3 | | | 10.81 | -5.99, 27.60 | | | *0.20* | |  | | 18.58 | 1.58, 35.57 | | | | *0.032* |  | | 31.11 | 13.62, 48.61 | *0.0005* |
|  | Quartile4 | | | 10.85 | -5.58, 27.29 | | | *0.19* | |  | | 25.95 | 9.86, 42.05 | | | | *0.0016* |  | | 35.48 | 17.41, 53.54 | *0.0001* |
|  | *P_trend_* ^2^ | | | *0.15* | | | | | |  | | *0.0016* | | | | | |  | | *<.0001* | | |
| *Beverage and fired food* | | | | |  | | |  | |  | | | | | | |  |  | |  |  |  |
|  | Quartile1 | | | 0.00 |  | | |  | |  | | 0.00 |  | | | |  |  | | 0.00 |  |  |
|  | Quartile2 | | | -7.24 | -23.39, 8.90 | | | *0.38* | |  | | -13.55 | -30.13, 3.03 | | | | *0.11* |  | | -19.71 | -36.60, -2.82 | *0.022* |
|  | Quartile3 | | | -12.72 | -28.13, 2.68 | | | *0.11* | |  | | -15.94 | -31.00, -0.88 | | | | *0.038* |  | | -22.56 | -41.00, -4.11 | *0.017* |
|  | Quartile4 | | | -6.57 | -22.93, 9.78 | | | *0.43* | |  | | -7.41 | -23.97, 9.14 | | | | *0.38* |  | | -5.72 | -24.18, 12.73 | *0.54* |
|  | *P_trend_* ^2^ | | | *0.28* | | | | | |  | | *0.29* | | | | | |  | | *0.38* | | |
| ^1^ Adjusted for age, BMI, education, smoking, alcohol consumption, residence, area, family history of fracture, serum vitamin D level, and glucocorticoid use >3 months | | | | | | | | | | | | | | | | | | | | | | |
| ^2^ Test for trend across quartiles from linear regression model  Abbreviations: BMD, bone mineral density; BMI, body mass index; CI, confidence intervals | | | | | | | | | | | | | | | | | | | | | | |

| Supplemental Table 5. Associations Between Dietary Patterns and Risk of Fractures Among Postmenopausal Women | | | | | | | | | | | |
| --- | --- | --- | --- | --- | --- | --- | --- | --- | --- | --- | --- |
| Dietary patterns | |  | Clinical fracture in the past 5 years (N=6676, case=369) | |  | Vertebral fracture (N=3198, case=364) | |  | Vertebral fracture grade 2 or above (N=3198, case=174) | | |
|  |  |  | OR^1^ | 95%CI |  | OR^1^ | 95%CI |  | OR^1^ | | 95%CI |
| *Carnivorous diet* | |  | | |  | | |  |  | |  |
|  | Quartile1 |  | 1.00 |  |  | 1.00 |  |  | 1.00 | |  |
|  | Quartile2 |  | 1.16 | 0.77, 1.74 |  | 0.58 | 0.36, 0.95 |  | 0.46 | | 0.23, 0.94 |
|  | Quartile3 |  | 0.88 | 0.56, 1.37 |  | 0.40 | 0.24, 0.68 |  | 0.44 | | 0.21, 0.93 |
|  | Quartile4 |  | 0.56 | 0.34, 0.92 |  | 0.29 | 0.15, 0.47 |  | 0.18 | | 0.07, 0.48 |
|  | *P_trend_* ^2^ |  | *0.022* | |  | *<.0001* | |  | *0.0016* | | |
| *Vegetarian diet* | |  | | |  | | |  |  | |  |
|  | Quartile1 |  | 1.00 |  |  | 1.00 |  |  | 1.00 | |  |
|  | Quartile2 |  | 1.07 | 0.71, 1.62 |  | 0.72 | 0.46, 1.20 |  | 0.74 | | 0.42 1.33 |
|  | Quartile3 |  | 1.13 | 0.75, 1.70 |  | 1.08 | 0.66, 1.77 |  | 1.01 | | 0.70, 2.11 |
|  | Quartile4 |  | 1.09 | 0.67, 1.76 |  | 0.51 | 0.32, 1.09 |  | 0.29 | | 0.15, 0.59 |
|  | *P_trend_* ^2^ |  | *0.63* | |  | *0.21* | |  | *0.12* | | |
| *Dairy, fruit, and egg* | | | | |  | | |  |  | |  |
|  | Quartile1 |  | 1.00 |  |  | 1.00 |  |  | 1.00 | |  |
|  | Quartile2 |  | 1.47 | 0.90, 2.41 |  | 1.19 | 0.72, 1.97 |  | 1.06 | | 0.49, 2.29 |
|  | Quartile3 |  | 2.71 | 1.65, 4.46 |  | 0.85 | 0.50, 1.43 |  | 0.88 | | 0.43, 1.78 |
|  | Quartile4 |  | 2.08 | 1.25, 3.46 |  | 1.04 | 0.59, 1.58 |  | 0.84 | | 0.34, 2.07 |
|  | *P_trend_* ^2^ |  | *0.0002* | |  | *0.69* | |  | *0.59* | | |
| *Beverage and fired food* | | | | |  | | |  |  | |  |
|  | Quartile1 |  | 1.00 |  |  | 1.00 |  |  | 1.00 | |  |
|  | Quartile2 |  | 0.90 | 0.58, 1.29 |  | 1.19 | 0.76, 1.86 |  | 1.07 | | 0.56, 2.02 |
|  | Quartile3 |  | 0.87 | 0.57, 1.35 |  | 1.25 | 0.78, 2.01 |  | 1.26 | | 0.62, 2.56 |
|  | Quartile4 |  | 1.27 | 0.80, 2.03 |  | 1.29 | 0.72, 2.31 |  | 1.55 | | 0.65, 3.72 |
|  | *P_trend_* ^2^ |  | *0.49* | |  | *0.36* | |  | *0.29* | | |
| ^1^ Adjusted for age, BMI, education, smoking, alcohol consumption, residence, area, family history of fracture, serum vitamin D level, and glucocorticoid use >3 months | | | | | | | | | | | |
| ^2^ Test for trend across quartiles from logistic regression model  Abbreviations: BMI, body mass index; OR, odds ratios; CI, confidence intervals | | | | | | | | | |  | |
